# ‘This is the country of premature old men’ Ageing and Aged Miners in the South Wales Coalfield, c.1880–1947[Fn FN0001]

**DOI:** 10.1080/14780038.2015.1088682

**Published:** 2016-03-24

**Authors:** Ben Curtis, Steven Thompson

**Affiliations:** ^a^ Cardiff University; ^b^ Aberystwyth University

**Keywords:** miners, ageing, disability, life-cycle, pensions

## Abstract

This article considers the effects of work in the south Wales coal industry either side of the turn of the twentieth century and, specifically, the ways in which work aged workers prematurely. It examines the consequences of working practices for miners’ bodies, the expedients utilized by miners to try and cope with the effects of premature ageing, and the consequences for their living standards, experiences and status. It situates these phenomena in the contexts of industrial relations and welfare provision. In so doing, the article engages with historiographies of the life-cycle, the aged, and pensions provision in modern Britain.

Early in 1936, the journalist Louise Morgan, writing in the *News Chronicle* newspaper, published a series of articles on the ‘scourge of silicosis’ that affected workers in different industries across Britain. In one of those articles, focused on the coal industry in south Wales where the problem was particularly acute, Morgan lamented the terrible toll taken by the industry on the health of too many of its workers and the effects of this pulmonary disease on men’s bodies. Referring to south Wales, she wrote ‘This is the country of premature old men. You see them everywhere – bent, shuffling, panting for breath at any age between 35 and 50.’^1^ In making this comment, Morgan echoed a trope that had been present in writings about the British coal industry for a century or more by the 1930s. This trope emphasized the harmful effects of work in the industry and also, more generally, stressed the unnatural character of industrialization and its consequences for society. Morgan utilized the idea of premature ageing in a skilful and effective attack on an industry that had gained a terrible reputation for the excessive risks faced by its workers and for the employers that seemed to care little for the consequences of those risks for the men they employed.

This trope of premature ageing appeared in various debates, discussions and commentaries by miners and their supporters to emphasize the deleterious effects of work in such a harmful industry. References to aged or prematurely aged miners were usually used in order to bring pressure to bear on employers or government to enact improvements to working conditions or additions to welfare provision; such references were often allied to a discourse relating to the moral economy of aged miners in which employers were reminded of their obligations to those men who had given so much to increase their profits. At the same time, of course, the idea that underground work had an impact on the health and fitness of miners was not some mere rhetorical device intended for political effect – it was firmly grounded in objective reality and everyday experience: work in the coal industry was indeed harmful to health and caused a massive amount of injury, impairment and disability during the modern period that aged men prematurely. Furthermore, the absence of realistic alternatives meant that elderly miners did not retire, they merely worked until their strength failed or disability prevented them from continuing in their employment.

This article considers ageing and aged miners in the coal industry in south Wales in the last quarter of the nineteenth century and early decades of the twentieth and, in doing so, contributes to a number of important historiographies.^2^ In the first place, the concept of the life cycle, and its implications for poverty and wellbeing, for daily experiences and working lives, and for welfare provision and medical care, is one that is familiar to many historians. Ever since Seebohm Rowntree employed the idea in his study of poverty in York at the turn of the twentieth century, social commentators and historians have been attuned to the ways in which events in the life cycle can cause individuals and families to fall into, or escape from, poverty during the course of their lives.^3^ A focus on miners, as an occupational group, subject to particularly hazardous working conditions, can help to reveal the idiographic character of the impact of stages in the life cycle on the economic status of families. In particular, miners in their forties, thirties and even twenties, who suffered debilitating chronic pulmonary disease in the 1930s and 1940s, were prematurely aged by the effects of pneumoconiosis and classed as ‘old’, ‘aged’ or ‘infirm’, in the same way as men in their 50s and 60s. This serves to complicate our understanding of the life cycle and emphasizes again that ‘old age’ possessed a more contingent, subjective quality.

Second, this attention to aged and ageing miners can contribute insights to the historiography of the aged in modern Britain.^4^ In this regard, historians and others have debated the social aspects of ageing and old age, the changing conception of old age over time, the elderly and work, the place of the elderly in the family, medicine, institutions, welfare provision, and so on. A study of miners, as workers in one of the most iconic industries of the industrial revolution, can help to provide a further insight into the varied experiences of the aged and prematurely aged in the past and, in particular, can show some of the contexts in which workers were marginalized by, or, alternatively, offered opportunities in, a particular industrial context. It can also help to demonstrate the factors that led to old age in the first place and it offers the possibility to explore cultural ideas and contemporary definitions of old age in the past in a particular context. In this sense, it can explore the extent to which words such as ‘old’, ‘aged’, ‘infirm’ and ‘disabled’ were used, at times, as synonyms for each other and used in different combinations, or interchangeably, in a variety of contexts. This can allow us to explore the imprecision over definitions of old age, in addition to the uncertain borderlines between impairment, ill-health and disability. It also allows us to consider some of the ways in which old age and premature ageing were used for polemical purposes in a particular social, political and cultural context.^5^

Third, the particular ways in which attempts were made to make provision for this particular occupational group allows a contribution to be made to the historiography of pensions and, more generally, welfare provision in modern Britain.^6^ In debates over the appropriate responses to ageing and aged miners can be found all manner of different views as to the responsibilities of individuals, employers and the state, and ideas about the extent to which such men were deserving of particular levels and forms of support.^7^ More than that, a study of miners in the early twentieth century provides another illustration of the ways in which pension provision was utilized by employers and trade unions in their industrial relations strategies.^8^ In these various engagements with different historiographies, therefore, it is possible to obtain a sense of the interactions of social structure and culture in the construction of perceptions and understandings of old age, ageing and retirement.

## The Causes of Premature Ageing

The idea of premature ageing was a prevalent one in industrial society and especially so in coal communities. Contemporaries and observers were very aware of the effect that arduous physical labour in the industry, particularly work underground, had on the miner’s body and the extent to which it aged men and shortened life. This awareness informed the popular culture of nineteenth-century Wales as ballad singers and poets perceived the ageing effects of work in the coal industry and as the idea of premature ageing became a staple in the various poems, ballads and laments on the miner’s lot. In ‘Cwyn y gweithiwr tanddaiarol’ (‘The Underground Worker’s Lament’), published in the 1850s, for example, the balladeer sung that:

Tybia rhai fi’n, bedwar ugain,
Pan nad wyf ond deg a deugain.^9^
[Some wonder if I am eighty years old,
When I am no older than fifty.]

while Ieuan Gwynedd, a poet, insisted:

Glo, glo, glo! sy’n cynnar difa’m nerth,
Nes ydwyf bron, yng nghanol oed,
Yn weithiwr hen di-werth.^10^
[Coal, coal coal! that destroys my strength prematurely,
Until I am almost, in middle age,
An old, worthless worker.]

The disproportionate effect of mining in this regard, relative to other occupations, was also recognized by another writer in these years: ‘Y mae pob dau weithiwr arall yn byw yn gyfartal i dri glöwr!’ [‘Every two other workers live the equal of three miners!’]^11^

Later in the century, and into the first decades of the twentieth, the idea of the ageing effect of work in the industry reoccurred on numerous occasions, all of them characterized by tension, disagreement and difficulty as the trope of premature ageing was taken up in various industrial relations contexts. In the discussions over the introduction of an eight-hour working day in the 1890s, for example, some miners’ representatives stressed that the conditions in the deeper mines of the late nineteenth century aged miners more rapidly than the shallower workings of earlier periods and so pressed the case for a shorter working day.^12^ In the same decade, when a scheme to provide old age allowances to members of the South Wales and Monmouthshire Permanent Provident Society was first mooted, many individuals pointed out that few miners reached the age of sixty-five, the proposed age at which the pensions were to be paid, and so the scheme needed to adjusted accordingly.^13^ In this context, the definition of ‘old age’ was disputed and the retirement age considered appropriate for other occupational groups was judged to be too old for miners.

Even for those outside the industry, coal mining seemed to age its workers to a greater extent than other industries. By the 1930s, as the occupational health of miners came to be studied and considered to a greater degree by epidemiologists and industrial hygienists, so again the extent of premature ageing was used to convey the effects of work in the industry. In her research on the health of old and retired miners in south Wales, Enid Williams introduced her study by commenting on the groups of old miners who gathered in the public park in Aberdare: ‘Anyone would notice that the majority of these men had aged before their time and that they were very short of breath and frequently coughed.’^14^ But the extent of industrially induced premature ageing was perhaps most skilfully communicated by the ‘miner-writer’ Bert Coombes in the mid-twentieth century. In a book published in 1945, Coombes described bumping into an old friend, Tom Evans, a collier, and his two brothers:

‘These here are my brothers,’ Tom Evans stated . . . ‘This one got a shop of his own and this one is a vicar or something up Birmingham way’ . . . I noticed . . . the contrast between Tom and his brothers. One was two years older than Tom, the other three years younger, yet Tom looked old enough to be their father. His frame was drained of every ounce of fat, his hands curved inwards like claws, and his shoulders had a stoop. Of course he had plenty of blue marks on his hands and face. . . . In their younger days, so Tom told me later, they were ‘as alike as three peas’. What a quarter of a century of hard manual labour can do to a man, even if he was as lucky as Tom!^15^

Therefore, the matter of prematurely aged miners remained at the forefront of debates over the costs paid by workers for the winning of coal and informed understandings of industry, culture and society.

Apart from the prematurely aged, there were, in addition, a great many aged miners who continued in employment well past the time that other workers might have ended their working days, and this, of course, is little different to the experiences of so many other poor and working-class men in nineteenth- and early twentieth-century Britain. Some of the most skilled, best-paid workers in the nineteenth century were able to make provision for their old age, through work-placed pension schemes arranged through their trade unions, friendly societies, or, in some cases, by their employers.^16^ Other workers, including miners, however, were insufficiently paid, were not able to make provision for retirement and too often found themselves having to work into their sixties and even seventies. As Enid Williams commented ‘a doctor told me that it would be difficult to find many retired colliers. Many of the miners were extremely dyspnoeic and old, but they still carried on with their work’; she was told by one doctor that ‘“They don’t retire; they just work until they drop.”’^17^ In a sense, ‘retirement’ was often used to denote the inability to continue working as a result of disability rather than the arrival at some age at which working was no longer allowed, and Williams described men in their thirties, forties and fifties as ‘retired’.^18^ The 1901 census recorded a total mining workforce in the counties of Glamorgan and Monmouthshire of 136,818, of whom 2,108 (1.5 per cent) were over the age of 65.^19^ Even when pensions of 5s. a week were introduced for miners through the Monmouthshire and South Wales Permanent Provident Society in 1899, and across Britain more generally in 1908, the point was made repeatedly that many older miners would prefer to continue working since their earnings tended to be far greater than the small sum of the weekly pension.^20^

Interesting here is the use of language in which various words, such as ‘debility’ or ‘infirm’, were used in numerous different contexts to describe men that were old, physically impaired or both. Indeed, formulations such as ‘old and infirm’ were used frequently to describe aged, injured and diseased miners, where no simple delineation could be made between these states, while the word ‘old’ seems often to have been used in descriptions of colliers on the basis of their physical condition rather than their age. Another contemporary use of language that demonstrates the overlap in meaning in these various terms is the frequent formulation that old or injured miners were ‘disabled from working’, as though disability was not a physiological experience but rather merely the inability to work.

That work in the coal industry aged men prematurely should not come as a surprise. The arduous physical labour inherent in coal mining inevitably had a significant effect on the physique and appearance of miners. Working shifts of ten to twelve hours in the nineteenth century or eight or nine hours by the twentieth century, underground, in cramped conditions, in a deleterious environment, and with hard physical labour to be done, took its toll. Numerous writers noted that miners’ bodies did not have an ounce of fat, such was the severity of the work, and that their faces tended to be drawn and hollow.^21^ The toll taken by the strenuous nature of the work done by miners was exacerbated by the frequent injuries they suffered. Underground work was hazardous and there were numerous ways in which miners could suffer accidents and experience minor or more serious injuries. Heavy machinery and tools, falls of roof or sides, fast-moving trams of coal, the use of explosives, and countless other perils were responsible for the relatively high rate of accidents and injuries in Britain’s coal mines. In the period from 1910 to 1914, for example, 16.5 per cent of coal miners were injured every year compared with 8.3 per cent of metal smelters, 5.3 per cent of railway workers, and 2 per cent of workers in the cotton industry.^22^ At that time, a little over 1,000 ‘serious accidents’ occurred each year in the South Wales Coalfield and roughly 30,000 miners received injuries that caused disability lasting seven days or more in each year.^23^ Furthermore, this seriously under-estimated the total number of accidents that was experienced. Many injuries were considered too minor a reason for miners to lose time or, more importantly, wages, while compensation payments were not made for the first three days of work absence, and many workmen continued to work despite what would seem quite serious and certainly very painful injuries in other contexts.^24^ Frequent ‘knocks’ served to impair miners’ bodies, restrict ease of movement, and age the body prematurely.

Rheumatism and arthritis, as well as various inflammation ailments such as ‘beat knee’, ‘beat hand’ and ‘beat elbow’, also affected miners and caused considerable levels of impairment. These were caused typically by damage to joint cartilage through the continuous pressing of miners’ joints against the hard coalface floor during the course of their work. It could also be exacerbated by working in the wet conditions that characterized many coalfaces in south Wales. Across Britain as a whole, there were more than 10,000 new cases of the ‘beat’ diseases diagnosed each year in the 1940s and the early 1950s.^25^ Individuals who were sufficiently debilitated by rheumatism sometimes had to be admitted to workhouse infirmaries. Although most of these miners tended to be older men, individuals in their twenties, thirties and forties were also occasionally admitted on this basis.^26^ Another form of physical debility that miners suffered came in the form of hernias and other such strain-related injuries. Although rarely fatal, hernias and other such injuries would have been a source of continual pain and debility, limiting miners’ physical capacities.^27^

The effect of occupational disease on the body was even more deleterious. Miners were prone to a number of different ailments but it was pulmonary disease that was the most debilitating. Pneumoconiosis, a condition in which the lungs become congested and damaged owing to inhalation of coal dust particles over a period of time, was particularly harmful. It is difficult to obtain a clear statistical picture of the incidence of pneumoconiosis in the nineteenth and early twentieth centuries, as its sufferers tended to be listed in medical records as having either asthma or ‘phthisis’ (tuberculosis). Initially referred to by the more narrowly defined term ‘silicosis’, it was not classified as a compensable industrial disease until the Various Industries (Silicosis) Scheme of 1928, an amendment to the Workmen’s Compensation Act. Once the more broadly defined term of ‘Coal Worker’s Pneumoconiosis’ was officially recognized in 1943, the certified incidence of this condition rose dramatically: in south Wales, the number of new cases increased from 418 in 1939 to 5,224 in 1945.^28^

Although pneumoconiosis could be found in every coalfield in Britain, it was particularly prevalent in south Wales, especially during the early to mid-twentieth century. South Wales accounted for over 89 per cent of all new cases of pneumoconiosis in Britain between 1939 and 1945.^29^ According to the official statistics, silicosis and pneumoconiosis were responsible for the death of 1,334 south Wales miners and the permanent disablement of 18,297 others between 1937 and 1948. These figures are probably an underestimate, as they are only those who had been officially certified as suffering from the industrial disease.^30^ Furthermore, the tendency of south Wales miners to begin work at the coalface at a younger age than in most other coalfields meant that the incidence of pneumoconiosis was particularly high amongst younger miners: 33 per cent of all pneumoconiotics in the Cynon Valley and 26 per cent in the Rhondda Valley were under forty years of age in the 1950s, compared to a British average of less than 15 per cent.^31^ One former miner recalled in 1973 that there were ‘several’ young men in his pit who succumbed to pneumoconiosis: ‘I know of one young man . . . he died at the age of 28 with dust . . . in the year of 1939–1940. The face I worked in I believe the biggest part of the men have passed away.’^32^

For most miners, the first signs that they had pneumoconiosis were the onset of coughing and breathlessness, along with a diminishing of their aerobic capacity and thus their ability to perform physical work; with pneumoconiosis in its early stages the effect on a person’s appearance was not always particularly obvious. In its latter stages, however, pneumoconiosis wrought a total physical deterioration upon its sufferers, with men finding they had to sleep propped in a sitting position because they did not dare to lie down, whose lungs had become almost solid, and who faced certain and painful death before too long. Even young miners who had formerly been strong and healthy were not spared the full extent of this devastating debility.^33^ By the time that a miner had reached this advanced stage of pneumoconiosis, the pulmonary debility and muscular weakness which it engendered had become overlaid with an additional manifestation of premature ageing, in which his physical appearance became radically altered by the scourging and wasting effects of the disease. Strong and robust men in the prime of their lives could wither away to emaciated shadows of their former selves within a comparatively brief timescale. Bert Coombes describes the condition of one such unfortunate individual with terrible clarity:

A man came to see me to-day. He moved in a slow stagger, . . . his speech was indistinct, and he paused after every word that he gasped out so that he could gain energy and breath. . . . He leant on a stick and swayed; and he trembled with that swaying. . . . He tried to work his fingers into a waistcoat pocket, and he made me think of an old man who seeks for a sweet to soften his lips; but this man was not old. Two years before I had been a close friend of his; then he had been about thirty-three years old, well-built, and healthy. I can count a dozen around here that are in similar state, but he is the nearest to death – in appearance – of them all. Surely he, who has seen so many of his mates die from silicosis, must know that he has not many more weeks to drag himself about.^34^

The impression given by the level of fatalities, the numbers of accidents and injuries, and the extent of occupational disease, communicates the dangerous nature of work in the coal industry, but statistics on deaths, accidents, disablements and occupational disease tend to be snapshots of a particular point in time and serve to diminish the real significance for an individual miner during the life course. This was illustrated effectively by the *Report of the Royal Commission on the Coal Industry* published in 1925. The report applied the then current accident and disablement rates to a notional group of one hundred miners who worked in the industry for a period of twenty years. The report claimed that:

At present rates of accident and disease, therefore, it may be expected that in twenty years, among the hundred men, in round figures, two will be killed, nine will suffer fracture of the head or limb or other serious injury, eight will contract nystagmus, and eight more ‘beat hand, knee or elbow inflammation of the wrist,’ a total of 27 out of the hundred who will suffer at one time or other from these more serious dangers. In addition, there will be amongst them during the period 353 cases of comparatively minor accidents, each disabling, however, for a period of more than seven days; that is to say, that each of the men on the average will incur an accident of that character about once in six years. . . . If a miner worked underground, as many of them do, for forty years and not twenty, these risks would, of course, be doubled.^35^

This theoretical supposition offers a more realistic appraisal of the risks faced by miners, not in any one year, but during the course of their working lives. Furthermore, the report was written as the understanding of pulmonary dust disease was beginning to improve and a better sense of the true extent of the problem coming to be appreciated. As such, had the report been written twenty years later, the cumulative effects of debilitating pneumoconiosis would have been added to this list of hazards and the proportion of disability would have been even greater.

Therefore, in these numerous ways, the coal industry injured, maimed, crippled and prematurely aged vast numbers of men and produced a massive amount of illness, impairment and disability. Zweig, in his 1948 study *Men in the Pits*, was shocked by the extent of disability in mining communities. One miner he interviewed told him ‘Can’t you see for yourself the number of disabled men with amputated legs or arms or fingers, or even blind, or with twisted spines or necks, or otherwise laid on their backs?’ For his part, Zweig agreed with this impression: ‘Nowhere else can you see the same relative numbers of disabled men as in a colliery village’.^36^

## Disability, Ageing and the Miner’s Life-Cycle

Miners who found their strength failing or their lung capacity diminished were placed in an unenviable position. Industrial disability amongst mineworkers had profound and multi-layered psychological effects: victims and their families had to deal with the trauma of diagnosis, along with the internalized mental consequences of curtailment and loss of employment, physical deterioration, and invariably, the deeper implications of dependency and loss of self-esteem. These effects were even more pronounced when the individual in question had become disabled at a relatively young age: as was noted rather bleakly, ‘[i]t is bad for the soul to be a broken man at forty.’^37^ Additionally, in the highly gendered world of South Wales Coalfield society, industrial disability would also have impacted on individuals’ sense of masculinity and identity, in many cases removing them from a male-dominated work environment and relocating them within the domestic sphere, as well as removing the ability of male workers to provide for their families in a material sense – to act out the ‘breadwinner’ role. Industrially induced premature ageing also eroded their own sense of manly self-esteem by taking away their physical strength, leaving them feeling emasculated as a result of no longer being able to do ‘man’s work’. As one miner with advanced pneumoconiosis commented, in his household ‘the wife fills [i.e. shovels] the [house] coal, so it is a case I have to live with it now and look after yourself the best way you can’.^38^

Some pneumoconiotic miners would have faced their situation with a mixture of stoicism and fatalism, such as two brothers mentioned in a 1945 *Picture Post* article, who ‘mention casually enough that their father died of silicosis at 46’; however, as the article noted, ‘it cannot be a pleasant thought for a man to live with if he has the disease himself.’^39^ Nevertheless, given the prevalence of pneumoconiosis in South Wales Coalfield communities in the early to mid-twentieth century, the sense of tragedy must have been both pervasive and inescapable. In an industry and a culture that assigned status and wages to physical strength and labour productivity, ageing miners suffered a lessening in status, changes in occupation, and a fall in income. All of this was abundantly clear to each and every miner, and the consequences of ageing preyed on the minds of these workers. Bert Coombes conveyed the helplessness of ‘the men who are beginning to age’:

They may have been twenty-five or thirty years underground, and if they had been in some jobs would have qualified for a pension. In the mines they feel their strength going and the limbs stiffening. The fear creeps into their consciences that their best days are gone; that even all their experiences will not counterbalance their weakening body. They guess their wage packet will get steadily smaller, and they have no reserves put away. It is a grieving realisation, and must cause a great deal of the bitterness which sours mining life nowadays. No hope for the future, no security for the family. The only prospect, when he cannot answer the call for work, is the miner’s pension – parish relief.^40^

Similarly, another miner, himself a sufferer of the ‘dread disease’ of pneumoconiosis, insisted that he and his fellow pneumoconiotics were the ‘living dead’ of the coalfield:

Slow and unsteady in step, the small hill leading to their homes leaves them exhausted . . . young in age, old in body, and wasted. With their bony frames and crumbling lungs, they trudge along to the end of their days . . . forgotten men.^41^

In such circumstances, as men aged prematurely, a number of expedients were utilized and these related to the particular character of mining’s occupational structure.^42^ Historians of the coal industry have noted the extent to which boys and young men in the collieries of Britain typically followed a similar occupational trajectory.^43^ Young boys of the age of ten in the mid-nineteenth century would start as ‘trappers’, opening the air-doors that regulated the flow of air around the underground workings, while, in the later part of the century and the early twentieth century, they would start employment underground at twelve or, later, fourteen years of age as assistants or ‘butties’ to colliers, often family members. As these boys grew in age and strength, so in their teens they would tend to become ‘putters’ in the earlier period, pushing trams of coal underground, or, later, ‘hauliers’, driving horses that pulled the trams. This rise up the occupational hierarchy also corresponded with a higher status and a better wage.

Subsequently, by their early twenties, these young men might reach the top of this occupational hierarchy and become colliers at the coalface, the dustiest part of the underground workings, cutting the coal from the face with a mandrel and loading trams by hand in cramped conditions. Crucially important here is the fact that the colliers who cut the coal were, in contrast to most other classes or workmen, paid by piece-rates. The socialist newspaper the *South Wales Worker*, in a trenchant analysis of the ‘infernal bustle of the piece-rate system’, asserted that it was responsible for ‘the tendency towards increased periods and possibilities of bad health and of shortening the length of life of the average miner underground’; the answer, it asserted, was the abolition of the ‘damnable piece-rate system’ which would mean that ‘the incentive to work at a murderously high pressure will be removed, and the abnormal expenditure of vital energies which saps the life and shortens the days of the average miner to-day, will no longer be maintained.’^44^ Thus, at the peak of their strength, as they also began to consider marriage and family life, colliers were incentivized to work faster and thereby increase their exposure to risk and the extent of the injury sustained by their bodies.^45^ The deleterious effects of work of this kind in this difficult environment meant miners’ bodies succumbed, sooner or later, to the arduous nature of the work and the injuries done by accidents and disease. Indeed, accident, disablement and occupational disease rates tended to be higher for colliers who cut the coal at the face than for other classes of workmen, thereby increasing the speed of this occupational trajectory for ageing and aged miners.^46^

As bodies began to age and fail, so miners were forced to attempt to cope with the difficulties. One response was regular absenteeism. Absenteeism came to be a contentious issue in the 1940s and, in the face of criticisms that miners selfishly neglected their duties and imperilled the war effort, Bert Coombes asserted that many miners missed a shift or two each week as they felt their chests tightening as a result of pneumoconiosis: they ‘hope to stave off the disease by losing time frequently and so clearing the lungs’.^47^ This was an expedient presumably used before the 1940s also. Ultimately, however, absenteeism was not a strategy that could be used for a prolonged period of time and the next step was for a miner to leave face-work and to move to another role in the pit. As Enid Williams observed, ‘Few coal miners go through their industrial lives from the very beginning to their retirement without a change of work’, and it might be argued that just as miners ascended to higher status, better pay and more arduous tasks during their teens and twenties so, in mirror fashion, did they experience another, opposite trajectory as their health and strength failed in their thirties, forties and fifties, and as the toll of working in the industry was felt on their bodies.^48^ In this sense, it is possible to map the occupational change experienced by workers in the coal industry on the life cycle and to discern a downwards occupational mobility as health failed and impaired bodies struggled with the demands of work.^49^

Colliers at the coalface tended to give up their work cutting coal and instead moved to labouring work underground, whether repairing roadways, labouring or doing any number of other, ‘lighter’ tasks.^50^ If the impact of premature ageing or the extent of disability was more pronounced, these colliers might have moved, or been moved, to the surface where they might have been put to work on the screens, picking pieces of stone out of the coal, or, where the deterioration in fitness was more pronounced, employed in the lamp room, filling lamps with oil or distributing them to colliers at the start of each shift.^51^ Those men who ended up on the screens perhaps returned to where they had first started work in the industry and might have found themselves in the company of boys or even women who were employed there, thus emphasizing their emasculation and loss of status.^52^ These various tasks of ‘light work’ had a much lesser status than the work at the face and tended to be paid at flat ‘day-rates’, which entailed a decrease in the weekly pay packet that a miner collected.^53^ Interestingly, American historians have described similar experiences and expedients as ‘partial’ or ‘gradual’ retirement, or else ‘retirement on the job’.^54^

## Retirement, Pensions and Labour Relations

The experiences of these ageing and aged miners, the expedients they utilized to minimize the impact on their earning capacity, and the measures taken to meet their particular needs were all affected by a variety of different social factors, cultural values and industrial considerations. Increasingly, retirement and pensions came to be considered more systematically and attempts were made to deal with the considerable problems that prematurely aged and aged miners posed to the industry and its communities. In the first place, the experiences of ageing and aged miners were affected by the attitudes and actions of their employers. Throughout the nineteenth century, a moral economy operated in which an employer was often judged by the ways in which he treated his aged employees. Anthony Hill, one of the ironmasters at Merthyr Tydfil, for example, gained a good reputation due to his payment of pensions to old, retired workmen in the 1830s and 1840s, while Cory Brothers Co. was also praised for the support it provided to aged and injured miners in the 1890s.^55^ In the same way, opportunities afforded to such old and injured workmen to carry on working, doing ‘light work’, were also commended.^56^ However, government intervention in the late nineteenth century, in the form of employers’ liability and workmen’s compensation legislation, changed the context in which this moral economy worked. Most importantly, many ‘old and infirm’ miners were dismissed by coal employers as a result of the passage of the Workmen’s Compensation Act in 1897. The legislation made these ‘old and infirm’ miners a ‘bad risk’ since it was believed that such individuals were more prone to accidents that could injure themselves and their fellow workers, thereby increasing the employers’ liabilities.

Very soon after the Act came into force in July 1898, large numbers of men – one estimate stated 1,000 of them – were dismissed by the coal employers in south Wales.^57^ Union leaders protested that coal companies had dismissed men in their forties and fifties, while the Poor Law Inspector also opined that employers seemed to consider men over the age of fifty to be too old to retain in employment.^58^ This happened in other regions and industries also, and it was perhaps the inevitable consequence of the particular terms of the legislation, but it is clear that an additional rationale acted in south Wales as the coal employers dismissed men for certain industrial relations purposes.^59^ Crucial here was the Monmouthshire and South Wales Permanent Provident Society, an organization that came into existence on 1 January 1881 in response to the Employers’ Liability Act of 1880. The Society paid disability benefits to members injured during the course of their work and death benefits to the families of members should they be killed. Significantly, membership involved ‘contracting out’ of the protection offered under the legislation. In effect, the Society was intended as a means by which employers avoided their responsibilities under the Act and were able to make less costly ‘mutual arrangements’ in an apparently joint venture with their employees; the Society proved extremely controversial and was the source of a great deal of tension between employers and workers for the duration of its existence.^60^

The Society established a new scheme as a result of the passage of the 1897 Workmen’s Compensation Act and this ran in parallel with the scheme in place before that piece of legislation was passed. Under the new scheme, allowed for under Section 3 of the Act, a number of enhanced or additional benefits were made available to the members, and at a lesser contribution rate, including an old age allowance. This enhanced scheme was clearly intended to meet the challenges posed by the 1897 Act and stave off the collapse of the Society as the miners were tempted to abandon it in favour of the benefits brought by the legislation. The old age allowance of 5s. a week was paid to the oldest miners, over the age of 65, who had been in membership for ten years or more, who were incapacitated by infirmity, and who were not in receipt of disablement benefit from the Society. The allowance was not paid as a right, therefore, and, instead, the Society undertook to devote any annual surplus to the payment of however many allowances that that surplus could sustain in the year. At the end of 1899, the first year in which an evaluation had taken place, the surplus on the fund was sufficient to pay 100 pensions and these were to be paid for the duration of the scheme, that is, to the end of 1903.^61^ Further surpluses allowed the granting of additional pensions in subsequent years so that a little over 400 pensions were paid, at a total cost of just under £20,000, by 1907, after which point the Society ceased to exist as a sizeable or significant organization.^62^

In 1898, following the long lockout of that year, and during which time the 1897 Act came into force, many ‘old and infirm’ were dismissed upon the return to work. William Abraham (‘Mabon’), the miners’ leader and Member of Parliament, insisted that this was part of a broader strategy by the coal employers to protect their interests and ensure their workers contracted out of the protection afforded by the Act. Mabon and his colleagues in the Rhondda valleys noted that certain employers refused to stop contributions from the workers’ pay packets for the old scheme under the Society on the one hand but were willing to collect the contributions for the new scheme that paid old age allowances on the other, whilst also dismissing the old workmen. This, Mabon and the other leaders insisted, was a cynical ploy to use these older workmen as a means by which to compel their fellow workers to contribute to the Society’s new scheme and thereby preserve the men’s status as standing outside the protection of the Act.^63^

These actions clashed strongly against the deeply ingrained moral economy of old age in this industrial society. Meetings were convened across the coalfield and the newly formed South Wales Miners’ Federation (SWMF) took the matter up as a test of its nascent strength. Walter Lewis, chairman of the Federation’s Rhymney Valley district, described the employers’ action as ‘tyrannical’ and stated that ‘These men had spent their strength, their substance, and their lives in the service of the company, and now they were cast adrift on the mercy of the world.’ For his part, Mabon, President of the new Federation, lambasted the industrialists who placed their profits before humanity and lamented ‘the old men who had been driven to the Workhouse through the cruel action of men in whose service they had spent the best part of their lives.’^64^

The Miners’ Federation was confronted with the fate of these older men as one of the first major challenges it faced and various districts of the union instituted levies of their members to raise funds from which payments were made to the men who lost their employment as a result of the 1897 Act.^65^ Weekly payments of 5s. were made to the men in this situation and by the end of 1898, 400 men were in receipt of the payment.^66^ Just as the employers utilized these old, disabled men in their industrial relations strategies, so did the Federation. Dai Watts Morgan, miners’ agent for the Federation’s Rhondda No.1 District, for example, stated that ‘The benefits that would be derived by the workmen under the Act, would, undoubtedly, re-pay them for any sacrifice they made to maintain this pension fund. It was true that other districts had dropped this fund, but that was no reason why the Rhondda District should not support those old warriors to the end of their days.’^67^ The benefits brought by the 1897 Act, therefore, were worth bearing the cost of the support of the old men who lost their employment as a result of the legislation, at least in the short term, as the miners’ leaders, and the labour movement more generally, campaigned for the introduction of a state pension.

The funds established by the Federation districts did not last more than a few years due to the burdens the required levies placed on members; the Rhondda No.1 District was one of the last districts to maintain a fund and it was abolished in August 1902.^68^ Subsequently, the Miners’ Federation threw its support behind the campaign for the introduction of state pension before that was eventually passed in 1908, and, in the following decades, attempted to bring about a supplementary pension for miners to go with the basic state pension.^69^ The context changed quite dramatically during the First World War as the greater demand for coal and the recruitment and, later, conscription of men into the armed forces provided greater opportunities for older men. In these circumstances, older, and even disabled, workers were viewed as a means to release men of military age for the front. At a recruiting meeting at Ebbw Vale in 1915, for example, Frederick Mills, managing director of the Ebbw Vale Steel, Iron and Coal Company, opined that ‘There was a large number of men available from the surface labour class whose places could be taken by older men, wounded men, and men who had gone into retirement, but who had come out to take their share.’^70^

The context in which older, perhaps disabled, men were able to secure employment in the coal industry changed again with the economic downturn of the interwar depression which hit the coal industry particularly hard, especially so in south Wales. One survey carried out in Merthyr Tydfil in 1929, for example, found that 60 per cent of the unemployed men in the town were over the age of forty and noted that a large proportion of them were ‘physically unfit’.^71^ Such men might have obtained employment in better times but were often the first to lose their jobs in the depression as employers had the opportunity to pick and choose their workers from among a large surplus of younger, fitter men. In such circumstances, the Miners’ Federation was limited to campaigning on pensions rather than attempting to exert pressure on employers to treat older workers more favourably. Annual conferences of the Federation throughout the 1920s included resolutions on the matter of pensions: the resolutions variously called for the state, the employers and the Miners’ Welfare Fund to make such provision for the workmen of the industry, but these had very little impact.^72^ From 1930, attempts were made to co-operate with the employers to establish a contributory pension scheme but these too came to naught.^73^ More significant developments took place in 1935 when employers and the Federation agreed to contribute sums of money to a fund but these efforts also failed and the Federation admitted defeat by early 1939 and determined to throw its support behind efforts to campaign for an increase in the state pension.^74^ Miners, similar to many other workers during the nineteenth and twentieth centuries, were reliant on poor relief or public assistance as their main source of support after they were no longer able to work, and the pension introduced in 1908 was far too little to allow anything other than a bare subsistence existence. It is therefore unsurprising that Zweig found in mining communities in the mid-1940s that ‘the old-age pensioners are often very bitter, feeling forgotten and forsaken by the community’.^75^

Significantly, the idea of premature ageing was not lost sight of in these discussions of pensions. While a retirement age of sixty-five or seventy was suggested by many, there were occasional assertions that, due to the arduous nature of work in the industry, miners should receive a pension earlier than other occupational groups. Many miners and their representatives, especially in the nineteenth century, before sixty-five became the widely accepted retirement age, thought that sixty-five was too old an age to set as the retirement age for miners, with one suggestion, in response to the proposed plan of the Permanent Provident society to offer pensions at sixty-five years of age, that there were many men of fifty-five who were more deserving and in need of a pension than other miners over the age of sixty-five.^76^ In such suggestions can be found a recognition of the ageing effects of work in the industry but these assertions also coincided with calls from within the labour movement that the age at which pensions were to be paid be lower than seventy or even sixty-five. The Trades Union Congress, the National Committee of Organised Labour and the Labour Representation Committee were all in favour of sixty-five as the pensionable age from the 1890s, while the Women’s Co-operative Guild believed sixty to be a more appropriate age. In the event, of course, the state pension was paid at seventy years of age when it was established in 1908 and this was only reduced to sixty-five in 1925.^77^

In other instances, and far more significantly, it was argued that, rather than set a retirement age, it would be far more appropriate for miners to receive a pension after a certain period of employment. Discussions within the Federation usually centred on sixty or sixty-five as the age at which miners should gain eligibility for any supplementary pension but, occasionally, other suggestions were made. The resolution passed at the annual conference in 1923, for example, held that all workmen with twenty-five years’ service should be paid a pension funded out of the proceeds of the industry.^78^ Similarly, writing in 1945, at a time when a pension scheme was being debated as part of the likely post-war welfare settlement, James Griffiths, MP for Llanelli and later Minister of Social Security in Attlee’s Labour government, suggested that miners should receive a pension after a period of forty years of labour in the industry.^79^ In the suggestion that miners should be paid a pension after a set period of employment in the industry, therefore, can be found the idea that miners could become prematurely aged as a result of their calling, and both required and deserved a pension earlier in their lives than the retirement ages considered appropriate for other occupational groups.

## Conclusion

A major issue in the historiography of ageing is the extent to which industrialization or modernization impacted on the function and status of the aged. Central to this process was the overwhelming tendency for certain aspects of the social status of working-class people to be defined in terms of their ability to work: inability to work inevitably entailed a significant reduction in an individual’s income and the increased possibility of dependency upon others, with both factors necessarily having a negative impact on elderly workers. Coal, as one of the iconic industries of industrialization, offers useful insights into this phenomenon, in various respects. A further complicating factor in such arduous physical working environments as coal mining was the strong probability that the nature of the work would of itself bring about some degree of premature ageing of its workforce, both via the cumulative toll of the work itself and also the effect of industrial injury and disease. The evidence from the coal industry in south Wales confirms the consensus in the historiography that ‘old age’ did not simply exist as an abstract concept but was instead overwhelmingly an experiential category mediated primarily by an individual’s physical well-being – with a whole series of occupational health risks embedded within the structure of the working environment operating continuously and having the aggregate effect of rendering a large section of the working population incapacitated and ‘old before their time’. In this industrial/labourist context, therefore, for functional purposes, ‘old age’ was the point at which miners could no longer work. For the coal miners of south Wales, ‘old age’ was often synonymous with ‘disability’, rather than simply having reached a certain number of years old.^80^

## Notes on Contributor

Ben Curtis is a Lecturer in Modern Welsh History at Cardiff University, specialising in the history of the South Wales Coalfield. He is the author of The South Wales Miners, 1964–1985 (published by the University of Wales Press in 2013), as well as a range of other articles and essays on the social, political, economic and cultural history of industrial south Wales. He is the Treasurer of Llafur, the Welsh People’s History Society, and also the recently-appointed General Editor for the South Wales Record Society.

Steven Thompson is a Senior Lecturer in the Department of History and Welsh History at Aberystwyth University with interests in the history of medicine, welfare, and disability. He has published widely on aspects of these issues in modern Welsh history, particularly in relation to the South Wales Coalfield, and is currently preparing a study of the history of medicine in south Wales from the late eighteenth to the mid-twentieth century.

## Notes

1. 
*News Chronicle*, 18 February 1936.

2. For a parallel study, of French miners in the nineteenth century, see Roland Trempé, ‘The struggles of French miners for the creation of retirement funds in the 19th century’, in Anne-Marie Guillemard (ed.), *Old Age and the Welfare State* (London, 1983), pp. 101–13.

3. On Rowntree and the life cycle, see Jonathan Bradshaw and Roy Sainsbury (eds), *Getting the Measure of Poverty: The early legacy of Seebohm Rowntree* (Aldershot, 2000); for studies that utilize the life cycle, see Tim Wales, ‘Poverty, poor relief and the life-cycle: some evidence from seventeenth-century Norfolk’, in Richard M. Smith (ed.), *Land, Kinship and Life*-*cycle* (Cambridge, 1984), pp. 351–88; Samantha Williams and Susannah Ottaway, ‘Life Course and Lifecycle: reconstructing the experience of poverty in the time of the Old Poor Law’, *Archives*, 23 (1998), pp. 19–29; Samantha Williams, *Poverty, Gender and Life*-*cycle under the English Poor Law, 1,760*-*1834* (Woodbridge, 2011); on the contingent nature of the life cycle, and indeed ‘old age’, see Michael Anderson, ‘The emergence of the modern life cycle in Britain’, *Social History*, 10, 1 (1985), pp. 69–87.

4. Jill S. Quadagno, *Aging in Early Industrial Society: Work, Family, and Social Policy in Nineteenth*-*Century England* (London, 1982); Margot Jeffreys (ed.), *Growing Old in the Twentieth Century* (London, 1989); Margaret Pelling and Richard Smith (eds), *Life, Death and the Elderly: Historical perspectives* (London, 1991); Pat Thane, *Old Age in English History: Past Experiences, Present Issues* (Oxford, 2000).

5. Thane, *Old Age in English History*, p.6.

6. Ann Shola Orloff, *The Politics of Pensions: A Comparative Analysis of Britain, Canada, and the United States, 1880*–*1940* (London, 1993); John MacNicol, *The Politics of Retirement in Britain, 1878*–*1948* (Cambridge, 1998).

7. Margaret Pelling and Richard Smith, ‘Introduction’ in Pelling and Smith, *Life, Death and the Elderly*, p. 25.

8. Thane, *Old Age in English History*, p. 251.

9. Ap Myfyr, *Cwyn y gweithiwr tanddaiarol* (Pontypridd, 1853).

10. Ieuan Gwynedd, ‘Cân y Glöwr’, in E. G. Millward (ed.), *Ceinion y Gân: Detholiad o Ganeuon Poblogaidd Oes Victoria* (Llandysul, 1983), p. 58.

11. 
*Baner ac Amserau Cymru*, 6 March 1861, p. 1.

12. 
*Mines (Eight Hours) Bill, Deputation to the Most Hon. The Marquis of Salisbury, K.G. and the Right Hon. Arthur J. Balfour, M.P., from the Miners’ Federation of Great Britain, 14th March 1895* (London, 1895), pp. 7–8.

13. 
*Brython Cymreig*, 30 August 1895, p. 2; *Evening Express*, 31 August 1895, p. 3.

14. Enid M. Williams, *The Health of Old and Retired Coalminers in South Wales* (Cardiff, 1933), p. 1; see also p. 40.

15. B. L. Coombes, *Miners Day* (London, 1945), pp. 52, 56–7.

16. It is entirely possible that the relatively high levels of owner-occupation in the South Wales Coalfield constituted a strategy on the part of workers to provide for their old age. One friendly society in the region advertized its mortgage provision with the slogan ‘No rent is better than a pension’; see West Glamorgan Archive Service, West Glamorgan District, No.43, of the Independent Order of Rechabites, *Twenty*-*fifth Annual Report and Balance Sheet for the year ending 31st December 1910* (Swansea, 1911), p.20. On working-class owner-occupation, see Steven Thompson, ‘“Conservative Bloom on Socialism’s Compost Heap”: Working-Class Home Ownership in South Wales, c.1890–1939’, in R. R. Davies and Geraint H. Jenkins (eds.), *From Medieval to Modern Wales: Historical Essays in Honour of Kenneth O. Morgan and Ralph A. Griffiths* (Cardiff, 2004), pp.246–63.

17. Williams, *The Health of Old and Retired Coalminers*, pp. 20, 39; a similar observation was made half a century earlier: Charles Booth, *The Aged Poor in England and Wales* (London, 1894), p. 246.

18. Williams, *The Health of Old and Retired Coalminers*, p. 41.

19. Census of England and Wales, 1901: County of Monmouth, pp. 50, 52; County of Glamorgan, pp. 58, 60.

20. 
*Cardiff Times*, 31 August 1895, p. 6; the author of this article pointed out that an old man over the age of sixty-five and employed in the lamp-station was unlikely to give up his weekly wage of 15s. in order to receive a pension of 5s.

21. Ferdinand Zweig, *Men in the Pits* (London, 1948), p. 5.

22. Arthur McIvor, *A History of Work in Britain, 1880*–*1950* (Basingstoke, 2001), p. 120.

23. Dot Jones, ‘Workmen’s Compensation and the South Wales Miner, 1898–1914’, *Bulletin of the Board of Celtic Studies*, 29, 1 (1980), p. 135.

24. For a good example, see B. L. Coombes, *I am a Miner* (London, 1939), pp. 69–70.

25. Arthur McIvor and Ronald Johnston, *Miners’ Lung: A history of dust disease in British coal mining* (Aldershot, 2007), p. 51.

26. For example, see the twenty-nine-year-old collier from Tir-y-Berth admitted to Merthyr Tydfil Workhouse in July 1929; Glamorgan Archives, UM/30/16, Merthyr Tydfil Union, Workhouse admission register, 1928–1931.

27. The surgical appliances most frequently supplied in the coalfield in this period were the various surgical trusses, abdominal belts and spinal corsets which were provided to help alleviate the symptoms of these conditions; for examples, see Truss and Wooden Leg Register, Dowlais Iron Company Employees, 1891–1902. Cresswell Family Practice Records, Dowlais (Glamorgan Archives, DX83/9/1).

28. McIvor and Johnston, *Miners’ Lung*, p. 56.

29. McIvor and Johnston, *Miners’ Lung*, p. 56.

30. The South Wales Area of the National Union of Mineworkers (NUM) contended that between 1937 and 1948 2,088 south Wales miners had died and 38,449 had been permanently disabled; NUM (South Wales Area) Executive Council Annual Report, 1948–9, p. 93.

31. McIvor and Johnston, *Miners’ Lung*, p. 56.

32. South Wales Miners’ Library (SWML), AUD164, John Morgan Evans interview, 13 June 1973. Also, young miners in the 1930s and 1940s were exposed to the worse dust problems brought about mechanization in a way not true of their predecessors; Williams, *The Health of Old and Retired Coalminers*, p. 45.

33. For insights on the symptoms, see *Picture Post*, 27 January 1945; John Morgan Evans interview.

34. Coombes, *I Am a Miner*, pp.67–8; see also Williams, *The Health of Old and Retired Miners*, p. 99.

35. 
*Report of the Royal Commission on the Coal Industry (1925), Vol. I., Report* [Cmd.2600], 1926, xiv, pp. 191–2.

36. Zweig, *Men in the Pits*, p. 6.

37. 
*Picture Post*, 27 January 1945.

38. John Morgan Evans SWML interview.

39. 
*Picture Post*, 27 January 1945.

40. B. L. Coombes, *Miners Day* (London, 1945), p. 41.

41. 
*The Miner*, January 1945, p. 14.

42. Following Margaret Pelling, ‘expedients’ rather than ‘strategies’ has been used here to reflect the limitations placed on the exercise of free choice; Margaret Pelling, ‘Old age, poverty, and disability in early modern Norwich’, in Margaret Pelling and Richard Smith (eds), *Life, Death and the Elderly: Historical Perspectives* (London, 1991), pp. 92–3.

43. Benson, *British Coalminers in the Nineteenth Century*, pp. 49–55.

44. 
*South Wales Worker*, 30 May 1914, p. 6.

45. It has long been recognized that, due to the relatively high wages that miners could earn from their early twenties, miners tended to marry earlier than men in other occupational groups: Michael R. Haines, ‘Fertility, Nuptiality, and Occupation: A Study of Coal Mining Populations and Regions in England and Wales in the Mid-Nineteenth Century’, *Journal of Interdisciplinary History*, 8, 2 (1977), pp. 259–62; B. L. Coombes, *Those Clouded Hills* (London, 1944), p. 26.

46. 
*Report of the Royal Commission on the Coal Industry 1925*, p. 192.

47. Coombes, *Those Clouded Hills*, p. 27, see also p. 29; see also Benson, *British Coalminers in the Nineteenth Century*, p. 59.

48. Williams, *The Health of Old and Retired Coalminers*, p. 96; Benson, *British Coalminers in the Nineteenth Century*, pp. 62–3. On a similar phenomenon in an American context, see Alan Derickson, ‘Occupational disease and career trajectory in hard coal, 1870–1930’, *Industrial Relations*, 32, 1 (1993), pp. 94–110; see also, Anderson, ‘The emergence of the modern life-cycle in Britain’, pp. 85–6.

49. This is a pattern recognised by other historians of work, old age and retirement in Britain: Paul Johnson, ‘The Employment and Retirement of Older Men in England and Wales, 1881–1981’, *Economic History Review*, New Series, 47, 1 (1994), p. 119; Pelling and Smith, ‘Introduction’, pp. 23–4; Thane, *Old Age in English History*, pp. 276–8.

50. Williams, *The Health of Old and Retired Coalminers*, p. 19; Williams notes that while an underground labourer’s job was less heavy than that of a collier, the work was often more dusty, thus tending to increase the possibility of contracting pneumoconiosis.

51. Enid Williams found that many colliers, unable to continue work at the coalface because of pulmonary disease, requested work on the surface and were often employed on the screens. In many instances, these screens were dustier than any other part of the colliery; Williams, *The Health of Old and Retired Coalminers*, p. 19–20.

52. Of interest here is Seth Koven, ‘Remembering and Dismemberment: Crippled Children, Wounded Soldiers, and the Great War in Great Britain’, *American Historical Review*, 99, 4 (1994), pp. 1,167-202.

53. Benson, *British Coalminers in the Nineteenth Century*, pp. 67–71.

54. Quadagno, *Aging in Early Industrial Society*, p. 21; Roger L. Ransom and Richard Sutch, ‘The Labor of Older Americans: Retirement of Men On and Off the Job, 1870–1937’, *Journal of Economic History*, 46, 1 (1986), pp. 1–30.

55. 
*Cambrian*, 11 June 1831, p. 2; *Monmouthshire Merlin*, 1 October 1842, p. 3; *Cambrian*, 28 December 1844, p. 3; *Pontypridd Chronicle*, 27 February 1891, p. 6.

56. For example, see *The Welshman*, 30 April 1897, p. 6.

57. 
*Cardiff Times*, 10 December 1898, p. 1.

58. 
*Cardiff Times*, 15 July 1905, p. 5; *Pontypridd Chronicle and Workman’s News*, 15 July 1905, p. 5; *Forty*-*first Annual Report of the Local Government Board* [Cd. 6,327, 6,331], 1912–13, xxxv, p. 133.

59. Quadagno, *Aging in Early Industrial Society*, p. 168; for a similar effect in America, see Nate Aldren, ‘Incentivizing safety and discrimination: Employment risks under workmen’s compensation in the early twentieth century United States’, *Enterprise and Society*, 15, 1 (2014), pp. 31–67. In fact, the miners’ leader William Abraham (bardic name ‘Mabon’) predicted that the dismissal of old workmen would be a consequence of the Act when the Bill was before the Commons and suggested that the introduction of old age pensions should be the answer to this; *Cardiff Times*, 22 May 1897, p. 1.

60. The permanent provident societies in other coalfields in Britain did not require members to ‘contract out’ of the protection afforded by the legislation as a condition of membership and so were more genuinely consensual in character; see John Benson, ‘Coalowners, Coalminers and Collaboration: The Miners’ Permanent Relief Fund Movement in England, 1860–1895’, *Labour History Review*, 68, 2 (2003), pp. 181–94; on the significance of ‘contracting out’, see Robert Asher, ‘Experience counts: British workers, accident prevention and compensation, and the origins of the welfare state’, *Journal of Policy History*, 15, 4 (2003), pp. 359–88, esp. pp. 362–5; also, P. W. J. Bartrip and S. B. Burman, *The Wounded Soldiers of Industry: Industrial Compensation Policy 1833*–*1897* (Oxford, 1983), pp. 158–73.

61. 
*Cardiff Times*, 5 May 1900, p. 6.

62. 
*Weekly Mail*, 6 February 1904, p. 9; *Cambrian*, 21 June 1907, p. 8. In this regard, the Monmouthshire and South Wales Society was far less successful than its counterparts in other coalfields. The Northumberland and Durham Miners’ Permanent Relief Fund not only offered pensions to men above sixty years of age (or at younger ages if a man was unfit for work) from 1874, but also provided subsidised housing for retired miners from the 1890s. See Thane, *Old Age in English History*, p. 245.

63. 
*Cardiff Times*, 17 December 1898, p. 6.

64. 
*Evening Express*, 18 November 1898, p. 2; *Cardiff Times*, 1 November 1898, p. 6.

65. 
*Evening Express*, 21 October 1898, p. 3.

66. 
*Evening Express*, 21 October 1898, p. 2.

67. 
*Weekly Mail*, 16 February 1901, p. 5; see also Watts Morgan’s comments in Monthly Report of the Rhondda No.1 District of the South Wales Miners’ Federation, 11 November 1901, pp. 10–11 (South Wales Coalfield Collection, Richard Burton Archives, Swansea University, SWCC/MNA/NUM/3/8/10a).

68. Monthly Report of the Rhondda No.1 District of the South Wales Miners’ Federation, 18 August 1902, pp. 4–9.

69. South Wales Miners’ Federation (hereafter SWMF), Minutes of the Annual Conference, 18–20 March 1907.

70. 
*Monmouth Guardian*, 5 November 1915, p. 3. The same rationale led Military Service Tribunals to deny claims for exemption from conscription on the basis that the work of the applicants could be done by older men; see, for examples, *Llais Llafur*, 1 April 1916, p. 1; *Cambrian Daily Leader*, 7 March 1916, p. 6.

71. National Library of Wales, Thomas Jones C. H. papers, John Davies and David E. Evans, ‘Report on Merthyr Tydfil’, 11 July 1929, p. 5.

72. For examples, see SWMF, Minutes of the Annual Conference, 22–24 June 1922; 18–20 June 1923; 25–27 June 1925; 1–2 July 1927.

73. SWMF, Executive Council minutes, 28 February 1930, 23 June 1930, 25 July 1930.

74. SWMF, Executive Council minutes, 31 January 1939; Thane, *Old Age in English History*, p. 251 notes that occupational pension schemes were fewest in number in industries with difficult industrial relations, insecurity of employment, and the payment of piece rates, and that in such cases, trade unions, unable to compensate members with union pension schemes, looked to the state for assistance. This was generally the case in relation to coal but even within the industry, south Wales was less able to make provision than its counterparts in other coalfields in which unions succeeded in establishing superannuation funds; see Ness Edwards, ‘The Miners’ Unions’, in G. D. H. Cole (ed.), *British Trade Union To*-*day: a Survey* (London, 1939), p. 290.

75. Zweig, *Men in the Pits*, p. 129.

76. For examples, see *Brython Cymreig*, 30 August 1895, p. 2; *Evening Express*, 31 August 1895, p. 3; *Cardiff Times*, 31 August 1895, p. 6.

77. Thane, *Old Age in English History*, pp. 14, 206, 208–9, 210.

78. SWMF, Minutes of the Annual Conference, 18–20 June 1923.

79. James Griffiths, *Glo* (Liverpool, 1945) p. 57.

80. Trempé, ‘The struggles of French miners’, p. 101.

